# Mutagenesis-Mediated Virus Extinction: Virus-Dependent Effect of Viral Load on Sensitivity to Lethal Defection

**DOI:** 10.1371/journal.pone.0032550

**Published:** 2012-03-19

**Authors:** Héctor Moreno, Héctor Tejero, Juan Carlos de la Torre, Esteban Domingo, Verónica Martín

**Affiliations:** 1 Centro de Biología Molecular “Severo Ochoa” (CSIC-UAM), Cantoblanco, Madrid, Spain; 2 Dpto. de Bioquímica y Biología Molecular I. Universidad Complutense de Madrid, Madrid, Spain; 3 Department of Neuropharmacology, The Scripps Research Institute, La Jolla, California, United States of America; 4 Centro de Investigación Biomédica en Red de Enfermedades Hepáticas y Digestivas (CIBERehd), Barcelona, Spain; 5 Centro de Investigación en Sanidad Animal (CISA-INIA) Instituto Nacional de Investigación Agraria y Alimentaria, Valdeolmos, Madrid, Spain; Institut Pasteur, France

## Abstract

**Background:**

Lethal mutagenesis is a transition towards virus extinction mediated by enhanced mutation rates during viral genome replication, and it is currently under investigation as a potential new antiviral strategy. Viral load and virus fitness are known to influence virus extinction. Here we examine the effect or the multiplicity of infection (MOI) on progeny production of several RNA viruses under enhanced mutagenesis.

**Results:**

The effect of the mutagenic base analogue 5-fluorouracil (FU) on the replication of the arenavirus lymphocytic choriomeningitis virus (LCMV) can result either in inhibition of progeny production and virus extinction in infections carried out at low multiplicity of infection (MOI), or in a moderate titer decrease without extinction at high MOI. The effect of the MOI is similar for LCMV and vesicular stomatitis virus (VSV), but minimal or absent for the picornaviruses foot-and-mouth disease virus (FMDV) and encephalomyocarditis virus (EMCV). The increase in mutation frequency and Shannon entropy (mutant spectrum complexity) as a result of virus passage in the presence of FU was more accentuated at low MOI for LCMV and VSV, and at high MOI for FMDV and EMCV. We present an extension of the lethal defection model that agrees with the experimental results.

**Conclusions:**

(i) Low infecting load favoured the extinction of negative strand viruses, LCMV or VSV, with an increase of mutant spectrum complexity. (ii) This behaviour is not observed in RNA positive strand viruses, FMDV or EMCV. (iii) The accumulation of defector genomes may underlie the MOI-dependent behaviour. (iv) LCMV coinfections are allowed but superinfection is strongly restricted in BHK-21 cells. (v) The dissimilar effects of the MOI on the efficiency of mutagenic-based extinction of different RNA viruses can have implications for the design of antiviral protocols based on lethal mutagenesis, presently under development.

## Introduction

RNA viruses display high mutation rates and frequencies that lead to the generation of dynamic mutant spectra termed viral quasispecies [1]–[8]. Quasispecies dynamics represents a serious impairment for the efficacy of antiviral treatments because of the rapid selection of viral mutants resistant to one or several antiviral agents. This problem has encouraged the search for alternative approaches to control viral infections. A new antiviral strategy termed lethal mutagenesis aims at increasing the viral mutation rate beyond a biologically tolerable threshold, resulting in reduced viral fitness and, eventually, virus extinction [9]–[14]. Arenaviruses are attractive pathogens to investigate lethal mutagenesis because of the very limited number of effective antiviral inhibitors available, and the impact of arenavirus diseases for human health [15]–[18].

Lymphocytic choriomeningitis virus (LCMV), the prototype arenavirus, is an enveloped virus with a bi-segmented negative strand RNA genome. Each genome segment uses an ambisense coding strategy to direct the synthesis of two viral polypeptides in opposite directions and separated by a non-coding intergenic region (IGR) [19], [20]. The L segment encodes the Z protein, a small RING protein that has matrix-like functions including budding activity and regulation of RNA synthesis [21], [22], and the RNA-dependent RNA polymerase or L protein [23]–[26]. The S segment encodes the glycoprotein precursor (GP-C) and the nucleoprotein (NP) [17]. Mutation frequencies within mutant spectra of molecular clones of LCMV replicated in BHK-21 cells are in the range of 1.0×10^−4^ to 2.7×10^−4^ substitutions per nucleotide [27], values which are comparable to those quantitated for other RNA viruses [28], [29].

In cell culture, the pyrimidine analogue 5-fluorouracil (FU) is mutagenic for LCMV, as evidenced by increases of the average mutation frequency of mutant spectra, which is associated with a decreased specific infectivity (PFUs per LCMV RNA molecule) [27], [30]–[34]. FU mutagenesis led to LCMV extinction both, during serial passages of the virus in BHK-21 cells, and upon extended, persistent infection in BHK-21 cells (infected cells maintained for a maximum of 100 h; no virus passage is involved [33], [30]). In both cases extinction occurred without any modification of the consensus nucleotide sequence of the population [31], [30], [27], [33], [32], [34]. LCMV decreased its intracellular and extracellular infectivity to a larger extent than the viral RNA levels [30]. This result, together with *in silico* simulations of the LCMV population dynamics, led to the formulation of the lethal defection model of virus extinction. This model proposes the participation of a class of altered RNA genomes termed defectors in the loss of infectivity [33], [30]. Defectors are RNA replication-competent genomes that encode altered versions of viral proteins that interfere with replication of the standard virus [30], [35], [36]. It must be underlined that, as defined, defectors differ from standard defective-interfering (DI) particles in that DI RNAs are dependent on standard virus for completion of their infectious cycle [37], whereas defectors may not depend on the standard virus.

DIs can be viewed as standard virus-dependent defectors. DIs of LCMV are rapidly generated during replication, and underlie homologous interference (also termed autointerference) in standard virus production [38]–[43]. In the description of our results we refer to defectors to mean any type of genome (dependent or not on the standard virus for replication) that has the potential to interfere with replication of the standard virus. The lethal defection model is congruent with data that indicate that interactions within mutant spectra may influence adaptation or de-adaptation of genome subpopulations [44]–[46], [36], [35], [47]–[49].

Studies with the picornavirus foot-and-mouth disease virus (FMDV) showed that low viral load and low fitness favored mutagen-induced virus extinction [50], [51]. In the present report, we have examined the effect of the initial viral dose on LCMV extinction, and found that following infection at low MOI, FU inhibited production of infectious progeny of LCMV, as well as of the rhabdovirus vesicular stomatitis virus (VSV), to a much larger extent than FMDV or encephalomyocarditis virus (EMCV). We propose that this different behaviour might relate to a favored generation of defector genomes during LCMV or VSV replication, as compared to the picornaviruses FMDV or EMCV. This interpretation is reinforced by the observation that passaged EMCV acquires an enhanced FU inhibition at low MOI. Given the increasingly recognized influence of the entire mutant spectrum in virus behavior [7], we present an extended lethal defection model that considers the effect that intracellular interactions among standard, mutant, and defector viruses can have on the extracellular virus dynamics. The model provides good support to the experimental results. Implications for antiviral therapy of the disparate effects of the initial viral load on the response of viruses to mutagenesis are discussed.

## Results

### Effect of 5-fluorouracil and the multiplicity of infection on lymphocytic choriomeningitis virus progeny production

**Figure 1:**
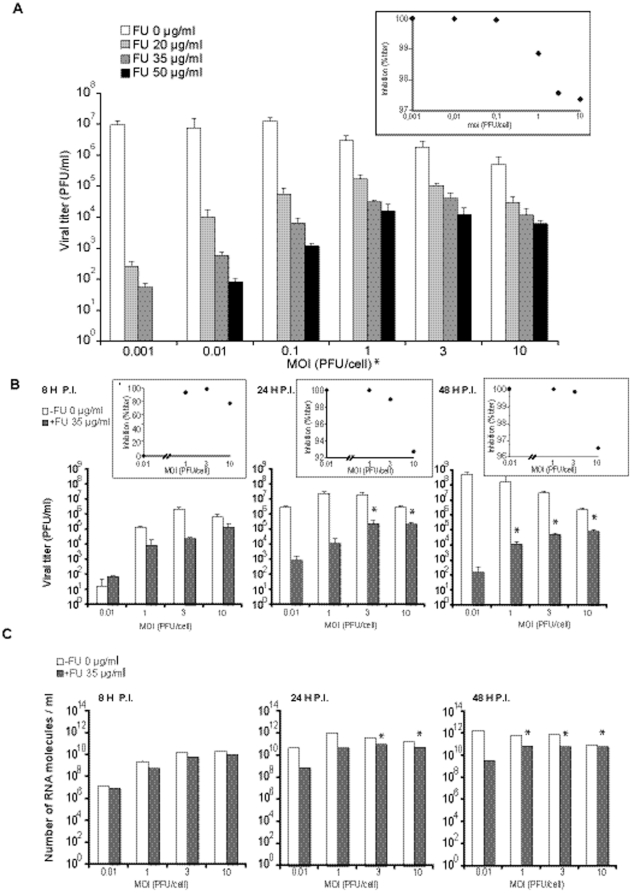
Effect of the multiplicity of infection (MOI) on the inhibition of LCMV production by 5-fluorouracil (FU). BHK-21 cells were infected with LCMV ARM 53b at the indicated MOI in the absence (white bars) or presence of different concentrations of FU (increasingly dark bars), as indicated in each panel. The asterisks above the bars indicate those inhibitions that are significantly different from those obtained in the infection at a MOI of 0.001 PFU/cell. A. Cell culture supernatants were harvested at 48 h p.i. and titrated. Viral titers are the average of at least four determinations and standard deviations are given. All pairwise comparisons with the inhibition at MOI 0.001 PFU/cell were significantly different. The inset visualizes the variation of the percentage of inhibition by 35 µg/ml FU as a function of the MOI. B. Similar to A, but with values for supernatants harvested at 8, 24 and 48 h p.i. Insets are as in panel A. C. Number of molecules of L genomic viral RNA in the cell culture supernatants harvested at 8, 24 and 48 h p.i. (same samples as those analyzed in B). RNA measures are the average of three determinations and standard deviations are given (low and not visible). Procedures are described in Materials and Methods.

Infections of BHK-21 cells with LCMV ARM 53b at MOIs of 0.001, 0.01, 0.1, 1, 3 and 10 PFU/cell, in the absence or presence of FU (20, 35, and 50 µg/ml), documented a dose-dependent inhibitory effect of FU at all MOIs tested, a decrease of progeny production as a function of the MOI in the passages in the absence of FU, and a decrease of inhibition by FU as a function of the MOI (Figure 1A). A general linear model analysis of the logarithm of the progeny production as a function of the logarithm of MOI and the inhibition by FU shows a significant interaction between the MOI and the inhibition by FU (p<0.05). Post Hoc multiple pairwise comparisons were carried out to compare the inhibition exerted by FU at the lowest MOI with that exerted at each of the other MOI for all the FU concentrations tested (p<0.05; pairwise t-test with Bonferroni Correction). This analysis revealed a significant decrease in the inhibition at the higher MOI for all the pairs tested. No significant differences were observed when comparing the differences in inhibition between MOI 3 PFU/cell and MOI 10 PFU/cell for all the concentrations of FU tested (p>0.05; pairwise t-test with Bonferroni correction).

**Figure 2:**
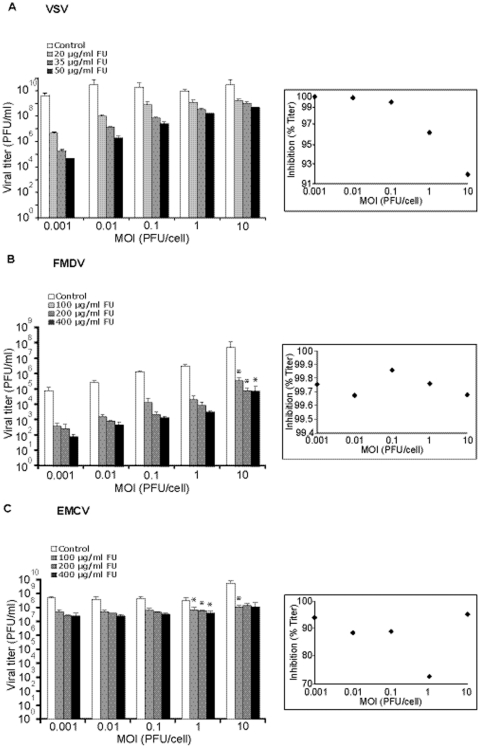
Effect of the multiplicity of infection (MOI) on the inhibition of vesicular stomatitis virus (VSV), foot-and-mouth disease virus (FMDV), and encephalomyocarditis virus (EMCV) by 5-fluorouracil (FU). BHK-21 cells were infected with VSV (A), FMDV (B) or EMCV (C) at the indicated MOI in the absence (white bars) or presence (increasingly dark bars) of different concentrations of FU, as indicated in each panel. Cell culture supernatants were harvested at 24 h p.i. and titrated as described in Materials and Methods. Viral titers are the average of at least four determinations and standard deviations are given. The asterisks above the bars indicate those inhibitions that are significantly different from those obtained in the infection at a MOI of 0.001 PFU/cell. No asterisks were added for VSV because all the pairwise comparisons with the inhibition at MOI 0.001 PFU/cell were significantly different. The panels on the right visualize the variation of the percentage of inhibition (by 35 µg/ml FU for VSV, 200 µg/ml FU for FMDV and EMCV) as a function of the MOI.

The negative correlation between the inhibition of LCMV production by FU and the MOI was observed when the virus titer was measured at 24 h and 48 h p.i., but not at 8 h p.i. (Figure 1B). Also, the decrease of infectious LCMV progeny production in the absence of FU when the MOI increased was observed at 48 h p.i. but not at 8 h or 24 h p.i. (Figure 1B). The effect of MOI on progeny production of LCMV RNA was less pronounced than the effect on infectivity, and the differences between RNA progeny at different MOIs did not reach statistical significance (Figs. 2B and 2C). Thus, both production of infectious LCMV progeny and the effect of FU on such a production depend on the MOI and the time p.i. at which the amount of progeny is determined.

### Effect of 5-fluorouracil and the multiplicity of infection on the replication of other RNA viruses

To investigate whether the decreased inhibition of LCMV infectious progeny production by FU at high MOI could be a more general phenomenon relevant also to other RNA viruses, we carried out experiments with VSV, FMDV and EMCV (Figure 2). The range of FU concentrations used in the experiments with FMDV and EMCV was increased relative to the concentration used with LCMV or VSV because of the lower sensitivity of these picornaviruses to the inhibitory activity of FU [50], [52], [53]. As with LCMV, the inhibition of VSV progeny production by FU decreased with the MOI (Figure 2A). A general linear model analysis evidenced a significant interaction between the logarithm of the MOI of VSV and the inhibition by FU (p<0.005). Post Hoc multiple pairwise comparisons showed that the difference in the inhibition, relative to the inhibition at MOI of 0.001 PFU/cell, was always statistically significant for all MOI and FU concentrations tested (p<0.05; pairwise t-test with Bonferroni Correction). As in the case of LCMV, no significant differences in inhibition were observed when comparing the inhibitions at MOI 1 PFU/cell and MOI 10 PFU/cell for all FU concentrations tested (p>0.05; pairwise t-test with Bonferroni correction).

**Figure 3:**
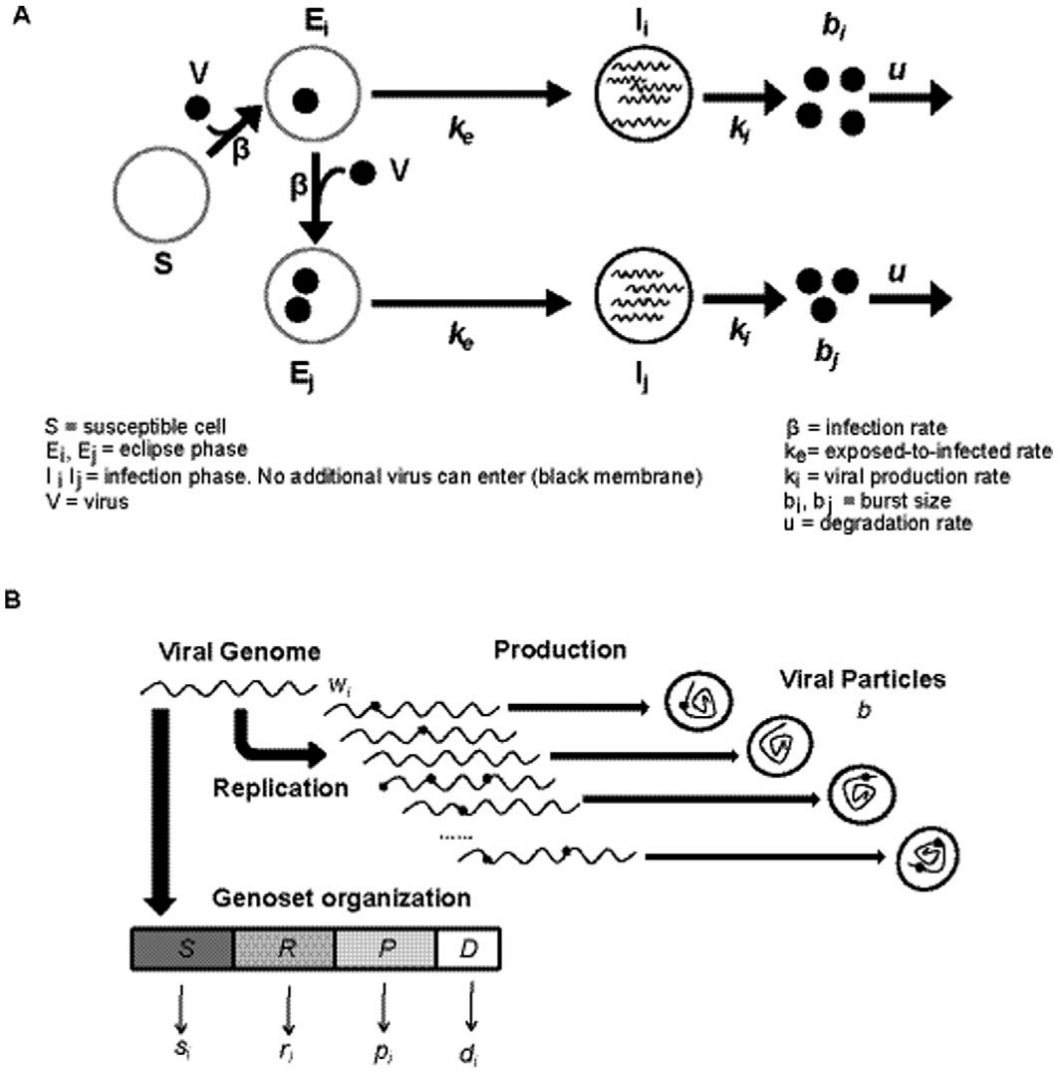
Basic features of the genosets model of lethal defection. A. Scheme of the infection of susceptible cells (S) by LCMV (V), stages of the infection process, and main parameters involved in the model (further detailed in the text and in Text S1). B. Scheme of the intracellular dynamics (excluding transcription) with formation of mutant genomes (dots on wavy lines) that may or may not be encapsidated. The boxes depict the genoset organization, which does not fit the physical map of the LCMV genome (see text), and which forms the conceptual basis of the extended lethal defection model presented here.

In contrast to LCMV and VSV, FMDV and EMCV did not display any global trend towards decreased inhibition by FU as a function of the logarithm of the MOI over the range of MOI tested (p = 0.325 and p = 0.517, respectively; General Lineal Model) (Fig. 3B and 3C). However, for FMDV a significant difference between the inhibition exerted by FU in the infections at MOI of 10 and MOI 0.001 PFU/cell was observed; for EMCV the difference was significant when comparing the MOI of 1 PFU/cell with 0.001 PFU/cell (p<0.05 in both cases; pairwise t-test with Bonferroni correction; Fig. 3B and 3C). Thus, a negative correlation between the inhibition of viral progeny production by FU and the MOI is not a general occurrence for RNA viruses, since it is clearly detected for LCMV and VSV, but not for FMDV and EMCV (Figs. 2 and 3).

### Genetic analysis of the 5-fluorouracil-treated viral populations obtained at different multiplicities of infection

Extended viral replication often results in an increase of the complexity of mutant spectra of viral populations [4], [7]. To investigate whether the amount of progeny virus correlated with genetic heterogeneity, we determined the complexity of the mutant spectra of LCMV populations, as well as values of progeny infectivity and progeny RNA and specific infectivity at 48 h p.i., following infection in the absence or presence of FU, at a MOI 0.01 PFU/cell and 10 PFU/cell (Table 1). The specific infectivity of progeny virus was about 10-fold lower in infections carried out at high MOI relative to low MOI, in the absence of FU. In contrast, the specific infectivity was about 30-fold higher at high MOI in the presence of FU. For all comparisons, the mutation frequency was significantly higher in the presence of FU than in its absence (p<0,05; χ^2^ test), as expected from the mutagenic activity of FU on LCMV [30], [31], [27], [32]–[34]. Interestingly, the mutation frequency of populations passaged in the presence of FU was 1.8-to 2.4-fold higher at a MOI of 0.01 PFU/cell than at a MOI of 10 PFU/cell, a difference that was statistically significant (p = 0.047; χ^2^ test). The increase of Shannon entropy in the populations passaged in the presence of FU was at least 1.6-to 6.4-fold higher at low MOI than at high MOI, mirroring the effect of the MOI on the mutation frequency (Table 1). In all cases, a decrease in specific infectivity was accompanied by an increase of mutant spectrum complexity (compare columns 5, 6 and 7 in Table 1).

**Table 1:** Quasispecies analysis of FU-treated LCMV populations.

5-Fluorouracil concentration (µg/ml)^a^	Gene analyzed^b^	MOI (PFU/cell)^a^	Progeny infectiviy (PFU/ml)^c^	Progeny RNA(molec/ml)^d^	Specific Infectivity(PFU/RNA molec)^e^	Mutation frequency (sn/nt)^f^	Shannon Entropy^g^	Sequenced nucleotides (nts)	Mutations Types				
									Transitions				Transversions^h^
									A→G	G→A	C→U	U→C	C→A
**0**	**L**	**0.01**	(5.6±1.7)×10^8^	(2.0±0.01)×10^12^	2.7×10^−4^	1.8×10^−4^	0.13	16,800	0	1	1	1	0
**0**	**L**	**10**	(2.5±0.6)×10^5^	(1.0±0.01)×10^11^	2.3×10^−5^	3.8×10^−4^	0.27	15,680	4	0	0	0	1
**0**	**Z**	**0.01**	(5.6±1.7)×10^8^	(2.0±0.01)×10^12^	2.7×10^−4^	<1.2×10^−4^	0	7,800	0	0	0	0	0
**0**	**Z**	**10**	(2.5±0.6)×10^5^	(1.0±0.01)×10^11^	2.3×10^−5^	2.5×10^−4^	0.10	7,800	0	0	0	1	1
**35**	**L**	**0.01**	(1.7±2.1)×10^2^	(3.7±0.01)×10^9^	4.5×10^−8^	2.2×10^−3^	0.83	16,144	28	0	0	5	0
**35**	**L**	**10**	(8.7±2.5)×10^4^	(6.6±0.01)×10^10^	1.3×10^−6^	9.0×10^−4^	0.44	14,325	12	1	0	1	0
**35**	**Z**	**0.01**	(1.7±2.1)×10^2^	(3.7±0.01)×10^9^	4.5×10^−8^	1.8×10^−3^	0.46	7,800	2	1	0	11	0
**35**	**Z**	**10**	(8.7±2.5)×10^4^	(6.6±0.01)×10^10^	1.3×10^−6^	1.0×10^−3^	0.25	7,800	0	0	0	7	1

a BHK-21 cell monolayers were infected at the indicated MOI in the absence or presence of 5-fluorouracil, as detailed in Materials and Methods.

b The genomic regions sequenced were the entire Z-coding region and residues 3654 to 4260 of the L-coding region.

c Titer of LCMV progeny determined at 48 h p.i.

d Quantification by real time RT-PCR of LCMV RNA in progeny, extracellular virus at 48 h p.i. in molecules/ml.

e Ratio of virus titer and number of viral RNA molecules per ml of cell culture supernatant determined at 48 h p.i.

f Average number of mutations per nucleotide, counted relative to the corresponding consensus nucleotide sequence.

g Proportion of different genomic sequences in the mutant spectrum of the quasispecies, calculated as described in Materials and Methods.

h The omitted transversion types were not represented in the sequences analyzed.

Unexpectedly, the bias in mutation types associated with FU mutagenesis differed between the L- and Z-genomic regions analyzed. A→G transitions were the most frequent mutation types in L, while U→C transitions were the most frequent types in Z (Table 1), and the difference was statistically significant (p<0.001; χ^2^ test). This genome segment-dependent mutational bias is under investigation.

The variation of mutant spectrum complexity as a result of FU mutagenesis in the course of infections with VSV, FMDV and EMCV at different MOIs showed dissimilar patterns. VSV displayed a behaviour similar to LCMV, but none of the differences reached statistical significance (Table 2). Under the conditions of MOI in which LCMV and VSV manifested a higher sensitivity to FU inhibition, its corresponding mutant spectrum displayed a higher complexity. The predominant mutation types induced by FU in VSV were A→G transitions. FMDV and EMCV showed the opposite trend than LCMV or VSV regarding the effect of FU or the MOI on mutant spectrum complexity, but none of the differences reached statistical significance (Table 2). Thus, these two picornaviruses deviate from the behaviour of LCMV regarding the effect of FU and the MOI on mutant spectrum complexity.

**Table 2:** Quasispecies analysis of FU-treated VSV, FMDV and EMCV populations.

5-Fluorouracil concentration(µg/ml)^a^	Virusanalyzed^b^	MOI(PFU/cell)^a^	Progeny infectiviy(PFU/ml)^c^	Mutation frequency (sn/nt)^d^	Shannon Entropy^e^	Sequenced nucleotides (nts)	Mutations Types								
							Transitions				Transversions^f^				
							A→G	G→A	C→U	U→C	C→A	A→C	U→G	G→U	G→C
**0**	**VSV**	**0.01**	(2.7±4.1)×10^10^	6.6×10^−5^	0.05	30,060	0	1	0	0	0	0	1	0	0
**0**	**VSV**	**10**	(2.6±4.1)×10^10^	6.2×10^−5^	0.05	32,064	1	0	0	1	0	0	0	0	0
**0**	**FMDV**	**0.001**	(8.5±4.8)×10^4^	1.3×10^−4^	0.10	23,715	1	0	0	2	0	0	0	0	1
**0**	**FMDV**	**1**	(3.3±0.9)×10^6^	3.9×10^−5^	0.02	25,296	1	0	0	0	0	0	0	0	0
**0**	**EMCV**	**0.001**	(4.5±1.4)×10^8^	1.9×10^−4^	0.15	30,720	2	0	2	2	0	0	0	0	0
**0**	**EMCV**	**1**	(2.8±2.0)×10^8^	3.4×10^−5^	0.02	29,440	0	1	0	0	0	0	0	0	0
**35**	**VSV**	**0.01**	(1.3±0.1)×10^7^	4.7×10^−4^	0.41	32,064	9	3	2	1	0	1	0	0	0
**35**	**VSV**	**10**	(9.9±3.9)×10^8^	2.5×10^−4^	0.21	31,396	5	1	1	1	0	0	0	0	0
**200**	**FMDV**	**0.001**	(2.7±2.7)×10^2^	1.9×10^−4^	0.13	25,296	2	1	2	0	0	0	0	0	0
**200**	**FMDV**	**1**	(9.2±6.2)×10^3^	4.5×10^−4^	0.29	22,134	1	0	1	7	0	0	0	1	0
**200**	**EMCV**	**0.001**	(2.5±0.2)×10^7^	1.9×10^−4^	0.15	30,720	1	0	3	1	1	0	0	0	0
**200**	**EMCV**	**1**	(5.4±0.8)×10^7^	2.3×10^−4^	0.18	30,080	1	1	1	3	0	0	0	0	0

a BHK-21 cell monolayers were infected with VSV, FMDV or EMCV at the indicated MOI in the absence or presence of 5-fluorouracil, as detailed in Materials and Methods.

b The genomic regions sequenced were nucleotides 5902 to 6569 of the L-coding region of VSV, 6800 to 7350 of the 3D (polymerase)-coding region of FMDV, and 6718 to 7403 pf the 3D (polymerase)-coding region of EMCV.

c Titer of VSV, FMDV and EMCV progeny determined at 24 h p.i.

d Average number of mutations per nucleotide, counted relative to the corresponding consensus nucleotide sequence. The mutation frequency of VSV increased in the presence of FU, and was 1.9-fold higher at low than at high MOI, but the differences were not statistically significant (p = 0.33; χ^2^ test).

e Proportion of different genomic sequences in the mutant spectrum of the quasispecies, calculated as described in Materials and Methods. Variations in Shannon entropy followed, the same trend as variations in mutation frequency but, again, the differences were not statistically significant (see footnote d).

f The omitted transversion types were not represented in the sequences analyzed.

### An extended lethal defection model

For a better understanding of the viral dynamics involved in lethal mutagenesis of LCMV, and to help in the interpretation of the experimental results, an extension of the lethal defection model [30], [54], [46], [35] has been developed. The extracellular virus dynamics follows the basic model of viral dynamics [55]. It is assumed that the cell can be in three different states: susceptible (*S*), exposed (*E*), and infected (*I*) (Figure 3A). One virus particle can infect an *S* cell, which becomes an *E* cell. The maximum number of particles that can enter a cell is determined by the parameter *maxVirusxCell* (see Table S1 for a list of parameters and their numerical values). Thus, the model takes into account different degrees of cell coinfection. Viruses are assumed to have the same internalization rate, *β*, for *S* and *E* cells due to an excess of receptor molecules on the cell surface [56]. Cells are in the *E* state during early times after virus penetration, prior to becoming *I* cells with a rate constant *k_e_*. New virus particles can enter *E* cells but not *I* cells. *I* cells release *b* infectious progeny with a rate constant *k_i_*. Since LCMV induces a persistent, non-cytolytic infection, the cell population (confluent monolayer) is taken as constant during the simulation, i.e. no cell death or cell growth are considered. Viruses are degraded according to a constant rate *u* (Figure 3A). Modeling of the intracellular dynamics has been based on our current understanding of the basic steps of arenavirus multiplication (Figure 3B). Three kinds of virus have been considered: (i) viable viruses that can complete an infectious cycle and produce infectious progeny by themselves; (ii) DI particles that cannot multiply in the absence of a viable virus, but that have a replicative advantage over the viable viruses when both coexist in the same cell [37], [57]–[59]. (iii) Defector viruses that are RNA replication-competent and can interfere with replication or production of the standard virus [30].

For simplicity, viral genomes are represented by four “functional genosets”. A functional genoset includes the genomic positions involved in a given viral function, irrespective of their location in the viral genome (Figure 3B). A given nucleotide can belong to more than one genoset but, as a first approach, this fact is not taken into account in this model. The effect of a genoset in a given viral function is expressed by a numerical value that depends on the number of mutations (relative to a given reference sequence) at any position within the genoset. Therefore, a viral genome is represented by a vector whose components are the mutations at each genoset.

Genoset *S* includes the residues that encode the exclusively *cis*-*acting* viral functions needed for the genome to be replicated. Genoset *R* involves the genome positions that participate in any viral function (needed for genome replication) that acts both in *cis* and *trans*. Genoset *P* includes the nucleotide positions involved in the production and release of viral particles. Genoset *D* encompasses any residue involved in the generation of DI particles. When the *D* genoset has zero mutations the genome is viable, and when the *D* genoset has at least one mutation the genome is a DI. Genosets *R* and *P* serve also to define two classes of defector genomes: the *R* genoset-associated defectors are those that can be replicated but do not contribute to the viral functions needed for genome replication; the *P* genoset-associated defectors are those that can be encapsidated but do not contribute to the production and release of viral particles. The mathematical functions relating the mutations in a given genoset, and their associated numerical functions are given in the Text S1.

Based on the four genosets, equations for the replication rate and burst size have been formulated. The model considers a single replication cycle. Replication of genome *i* produces a number of RNA copies (*w_i_*) per round of replication, which is given by the nearest integer number to 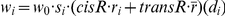, where w_0_ is the number of genomes produced by the standard LCMV genome, *s_i_* and *r_i_* are the numerical values associated to the *S* and *R* genosets, respectively, and *cisR* and *transR* are two parameters that permit assigning more weight to either *cis* or *trans* interactions in the functions associated with the *R* genoset; *cisR* and *transR* must fulfill the condition that *cisR*+*transR* = 1. The intracellular interactions among different viral genomes is introduced by means of the average replicative ability of the viral population, 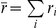, which is the average of the numerical values associated with all the *R* genosets of the genomes present in the same cell. *RepAdv* is a parameter that expresses the replicative advantage of DIs relative to standard and defector viruses. Thus, the replication ability *w_i_* of a genome depends on the intracellular viral genome population as a whole. During genome replication, each genoset mutates according to a Poisson distribution whose mean is the mutation rate per genoset (*U_S_, U_R_, U_P_*, and *U_D_*). The mutation rates per genoset are the product of the mutation rate per site, *m*, and the genoset length (see Text S1 and Table S1). A mutation rate per genome, *U*, is defined as the sum of the mutation rates per genoset.

Each cell can produce a maximum number of viral particles per unit time (*maxBsize*), which depends on the metabolic and physiological state of the cell. The total number of virus particles, infectious or not, *b*, produced by a cell, is given by 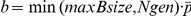, where *min(maxBsize, Ngen)* equals *maxBsize* when *Ngen* (which is the number of genomes inside the cell) is larger than *maxBsize* size; *min(maxBsize, Ngen)* equals *Ngen* when the latter is lower than *maxBsize*. To obtain the number of virus released from the cell, *min(maxBsize, Ngen)* is multiplied by the average efficacy of viral production in a cell, given by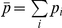 (a term equivalent to the average numerical values for all the *P* genosets present in the cell under consideration). Then, *b* genomes are selected at random and released from the cell. When *trans* interactions are not allowed, 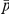 is taken as 1, and genomes are encapsidated according to the value of their *P* genoset.

An initial virtual virus population was obtained in a preliminary simulation consisting of a clone passaged ten times at a mutation rate (*U*) of 0.15 mutations per genome and replication round, a value which is used as the reference mutation rate throughout this study, based on average mutation rates determined for RNA viruses [60]–[62]. The initial population reached a mutation-selection balance [63] and a constant fraction of DI particles. A simulation begins with *c_0_* initial *S* cells that are infected at a given MOI (arbitrarily chosen between 0.01 and 3 PFU/cell) by the initial virus population, according to a Poisson distribution whose mean is the MOI. The infection continued until either the virus was exhausted or all the cells received the maximum number of virus particles allowed (*maxVirusxCell*). The *S* cells became *E* cells, and the simulation proceeded according to the parameters listed in the Text S1 and Table S1. An agent-based computational implementation of the model has been developed using MATLAB®. Details of the software will be published elsewhere and provided upon request.

### Computational simulations using the genosets model, and comparison with the experimental results

**Figure 4:**
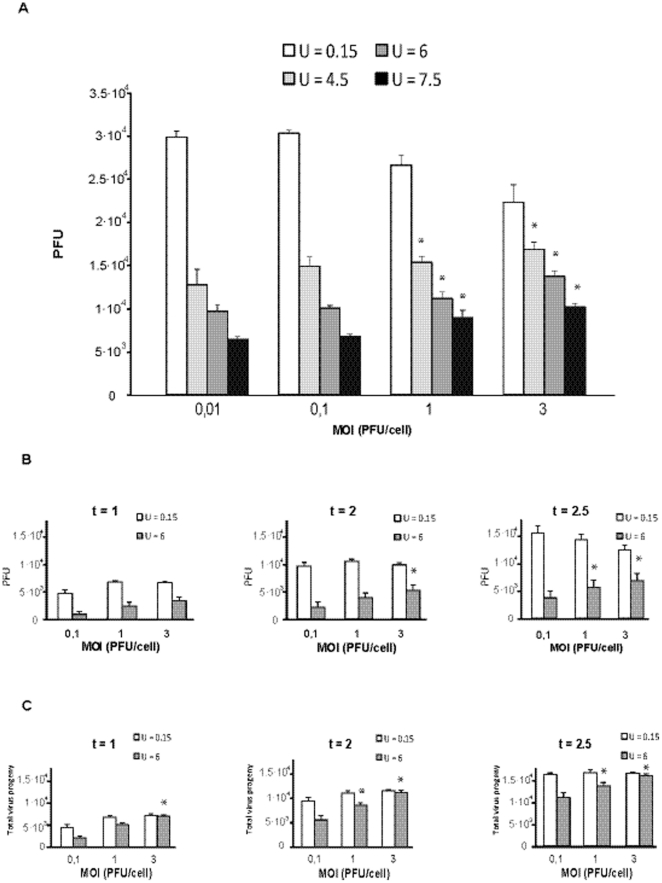
Viral production as a function of the multiplicity of infection (MOI) using the computational model developed in the present study (described in Materials and Methods and the Text S1). The asterisks above the bars indicate those inhibitions that are significantly different from those obtained at the lowest MOI in each simulation. A. Production of infectious virus (here termed PFU in the ordinate) at low (*U* = 0.15), medium (*U* = 4.5) and high (*U* = 6 and *U* = 7.5) genomic mutation rate (mutations per genome and round of copying) for different MOIs. B. Time dynamics of infectious virus production at different MOI and *U* = 0.15 and *U* = 6. Time is expressed in arbitrary units. C. Same as B but with infectious and noninfectious virus, equivalent to total virus progeny. The parameters used are listed in Text S1 and Table S1, but in the simulations performed in B and C the number of cells was reduced to 500. The results are the average of five simulations and the error bars are the standard deviations of those five simulations.

**Figure 5:**
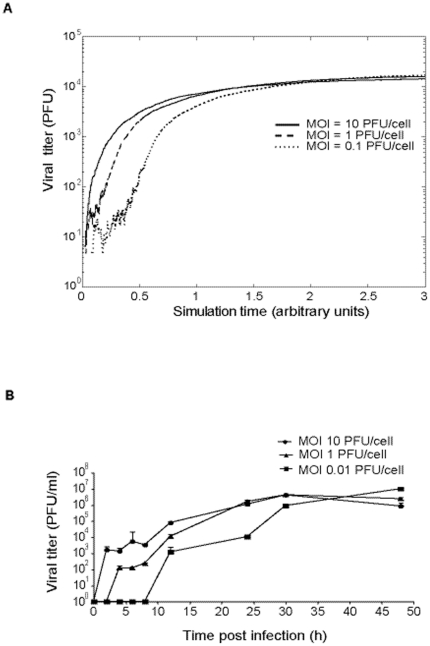
Kinetics of infectious progeny production as a function of the MOI, as predicted the theoretical model, and determined experimentally. A. Computational simulation of infections at MOI of 0.1, 1 and 10 PFU/cell. The parameters used are listed in Text S1 and Table S1. B. Experimental results: BHK-21 cells were infected with LCMV Arm53 at MOIs of 0.01, 1 and 10 PFU/cell, and supernatants were harvested at the indicated times postinfection and titrated. Viral titers are the average of at least four determinations and standard deviations are given. Procedures are described in Materials and Methods.

The computational model (described in Materials and Methods, with the invariant model parameters given in the Text S1 and Table S1) predicts that when the mutation rate *U* is 0.15 mutations per genome and replication round (m/g/r), infectious progeny production should decrease as the MOI increases because of homologous interference (autointerference) exerted by DI particles (Figure 4A). In contrast, the converse is predicted when *U* is increased to values of 4.5 to 7.5 m/g/r, (p<0.001, General Linear Model). These higher *U* values necessitate enhanced mutagenesis over the basal levels displayed by RNA viruses [62], [60], [61] (Figure 4A). When an arbitrary time factor spanning values of 1 to 2.5 was considered, the decrease in infectious progeny production with the MOI was only predicted at late times (t = 2.5) when *U* was 0.15 m/g/r. However, with a *U* of 6 m/g/r the infectious yield increased with the MOI at all times tested (t = 1, 2, 2.5) (Figure 4B). When the total extracellular progeny (infectious and non-infectious, taken as the total amount of viral RNA) was considered, an increase in total progeny production with the MOI was consistently observed taking a *U* of 6 m/g/r, but not a *U* of 0.15 m/g/r (Figure 4C). The model predictions regarding the effect of MOI and time of infection on progeny production (Fig. 5B and 5C) are consistent with the experimental results (Fig. 2B and 2C).

With a mutation rate *U* of 0.15 m/g/r, the model anticipates no significant differences among the mutation frequencies at the three MOI tested (p = 0.063; one-way ANOVA test), which is the result found with VSV in the absence of FU (Table 2). However, at *U* of 4.5 or 7.5 m/g/r the model predicts significant differences in mutation frequency at the three MOI tested (p<0.001, for both *U* = 4.5 m/g/r and *U* = 7.5 m/g/r; one-way ANOVA test) (Table 3). Post Hoc analysis shows that at both *U* of 4.5 m/g/r and *U* of 7.5 m/g/r, the mutation frequency at a MOI of 3 PFU/cell is significantly lower than the mutation frequencies at a MOI of 1 PFU/cell and 0.1 PFU/cell, (p<0.001 in all cases, Post-Hoc Bonferroni analysis); also, the mutation frequency at a MOI of 1 PFU/cell is significantly greater than the mutation frequency at a MOI of 0.1 PFU/cell (p<0.001 in all cases, Post-Hoc Bonferroni analysis), as found experimentally (Table 1). The theoretical model also predicted that the higher the MOI, the earlier the initiation of viral production (Figure 5A), and this was verified experimentally (Figure 5B).

**Table 3:** Computer simulations of the mutation frequency and percentage of inhibition of viral infectious progeny production, as a function of the multiplicity of infection (MOI) and of the *cis*- *trans* interactions.

MOI (PFU/cell)	Mutation frequency^a^	% Inhibition progeny production	Mutation frequency^a^	% Inhibition progeny production
	(mut/genome//mut/nt)	relative to U = 0.15^b^	(mut/genome//mut/nt)	relative to U = 0.15^b^
	*cisR = 0.25*		*cisR = 1*	
**Average number of mutations per genome, U = 0.15**				
**0,1**	1.09±0.52//7.3±3.5×10^−4^	-	1.88±0.92//1.3±0.6×10^−3^	-
**1**	1.41±0.65//9.4±4.3×10^−4^	-	1.55±0.34//1.0±0.2×10^−3^	-
**3**	1.63±0.09//1.1±0.1×10^−3^	-	1.68±0.16//1.1±0.1×10^−3^	-
**Average number of mutations per genome, U = 4.5**				
**0,1**	8.93±0.48//6.0±0.3×10^−3^	54±4	9.12±1.06//6.1±0.7×10^−3^	41±4
**1**	7.38±0.54//4.9±0.4×10^−3^	44±9	6.90±0.76//4.6±0.5×10^−3^	31±5
**3**	5.79±0.17//3.9±0.1×10^−3^	28±9	5.31±0.14//3.5±0.09×10^−3^	23±4
**Average number of mutations per genome, U = 7.5**				
**0,1**	12.57±0.59//8.4±0.4×10^−3^	77±2	11.21±0.94//7.5±0.6×10^−3^	60±5
**1**	10.59±0.21//7.1±0.1×10^−3^	68±1	9.26±0.47//6.2±0.3×10^−3^	50±3
**3**	8.52±0.11//5.68±0.07×10^−3^	53±4	7.59±0.09//5.1±0.6×10^−3^	43±2

a The mutation frequency is the average of ten simulations and is expressed as the number of mutations per genome (mut/genome) or mutations per nucleotide (mut/nt).

b The inhibition of infectious virus progeny production is calculated according to 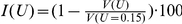, where, V(U = 0.15) is the number of viable progeny viruses (the equivalent of viral titer) when the mutation rate is U = 0.15 mutations/genome/replication round, and V(U) is the number of viable progeny viruses when the mutation rate is U = 4.5 or U = 7.5. Results are the average of ten simulations.

To explore to what extent the effect of MOI on infectious progeny production is affected by *cis-trans* interactions, simulations were carried out with the computational model either with no *trans* interactions allowed (*cisR = 1, transR = 0*), or with *cis* and *trans* interactions allowed (*cisR = 0.25; transR = 0.75*). A significant interaction between the MOI and the degree of *cis-trans* interactions, *cisR*, was observed both for U = 4.5 m/g/r and U = 7.5 m/g/r, (p<0,005 in both cases, General Linear Model) indicating that the lower *cisR*, the higher the effect of the MOI on infectious progeny production.

### Influence of prior mutagenesis of encephalomyocarditis virus on the multiplicity of infection-dependent inhibition by 5-fluorouracil

**Figure 6:**
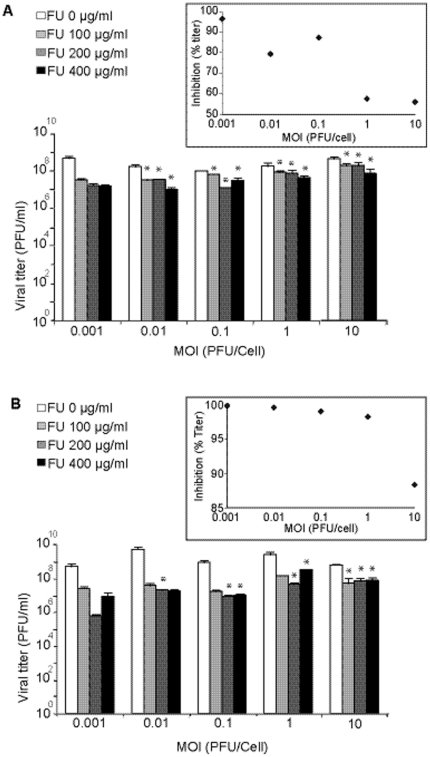
Effect of the multiplicity of infection (MOI) on the inhibition of mutagenized and non-mutagenized EMCV progeny production by 5-fluorouracil (FU). A. BHK-21 cells were infected with EMCV passaged 20 times in absence of ribavirin at the indicated MOI, in the absence (white bars) or presence of different concentrations of FU (increasingly dark bars), as indicated in each panel. Cell culture supernatants were harvested at 24 h p.i. and titered. Viral titers are the average of at least three determinations and standard deviations are given. The inset visualizes the variation of the percentage of inhibition (by 200 µg/ml FU) as a function of the MOI. B. Same experiment, but using a EMCV passaged 20 times in the presence of 800 µM ribavirin. The asterisks above the bars indicate the FU-inhibition values that are significantly different from those obtained with the corresponding lowest MOI tested. The inset is as in panel A. Procedures are described in Materials and Methods.

The different influence of the MOI on the inhibition of infectious progeny production by FU observed for different RNA viruses was intriguing (Tables 2 and 3). We considered that the different behavior of the viruses tested could be related to an easier production of DIs by LCMV and VSV than by FMDV and EMCV. Although DIs and other defective RNAs have been described for FMDV and EMCV [64]–[69], their interfering activity is generally less pronounced than that exerted by DIs of negative strand RNA viruses [70], [37], [59]. Since passages of virus increases the complexity of mutant spectra [4], [7], we investigated whether passage of EMCV could render the inhibition by FU MOI-dependent. The virus passaged 20 times in the absence or presence of ribavirin (R), a drug that is mutagenic for picornaviruses [71]–[73], showed a statistically significant increase of the inhibition by FU at low MOI relative to high MOI (p = 0.012, p = 0.047, respectively; General linear model) (Figure 6). The result implies also that the MOI dependence of the FU inhibition of progeny production can be influenced by a population context provided by the passage history of a virus.

### LCMV coinfections and reinfections in BHK-21 cells

**Figure 7:**
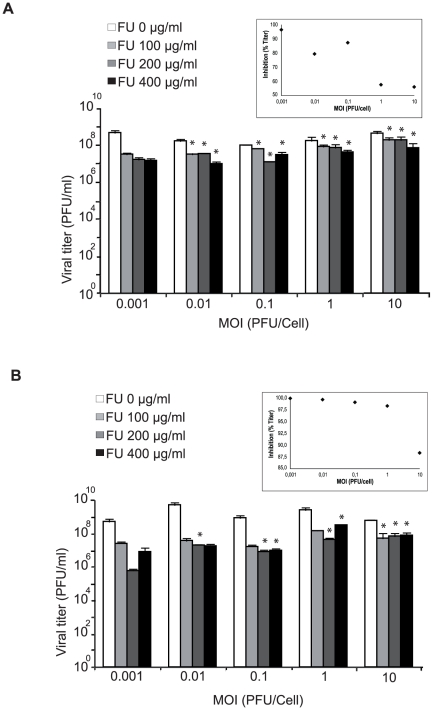
LCMV coinfections and reinfections of BHK-21 cells. Cells were infected with RFPrLCMV at the indicated MOI. At 0, 4, 8, 12 or 24 h p.i. cells were re-infected with GFPrLCMV at a MOI of 0.1 PFU/cell. The different panels show representative images of infected cells at 0 (A) and 48 (B) h p.i. with GFPrLCMV. Magnification is 20-fold. Nuclei were stained with DAPI. The origin of the marked RFPrLCMV and GFPrLCMV and procedures for the infections are described in Materials and Methods. Additional images of coinfections and reinfections at different MOI, and controls are depicted in Figures S1, S2 and S3.

Interference can be the result of mutated, defective genomes that act on infectious genomes contained in the same cell, or mutated genomes that are released from the cells where they are produced and then can reinfect other cells together or sequentially with standard infectious virus. To test whether a cell infected with LCMV could be reinfected by another LCMV (that is, that there is no superinfection exclusion that would preclude interference by externally added mutants), we used trisegmented versions of recombinant LCM viruses (r3LCMV) [74] expressing either RFP (r3LCMV/RFP) of GFP (r3LCMV/GFP). Cells were first infected with r3LCMV/RFP at MOIs of 0.001, 0.01, 0.1, 1 or 10 PFU/cell, and the infection monitored based on intracellular RFP expression. The first red cells could be detected at 24 h p.i. in the infections carried out at MOIs of 0.001, 0.01 or 0.1 PFU/cell, and at 8 h p.i. at a MOI of 1 or 10 PFU/cells (Figs. 7A and S1). At 0, 4, 8, 12 and 24 h after the first infection, the cells were infected with r3LCMV/GFP at a MOI of 0.1 PFU/cell. Replication of the second virus (green cells) was not observed in any case (independent on the time between both infections) in cells that had been infected with r3LCMV/RFP at high MOI (Figs. 7A and S1). In cells infected with r3LCMV/GFP at low MOI, replication of the second virus (r3LCMV/GFP) was observed when the time between infections was 12 h or less (Figs. 7B and S1). Thus, superinfections were heavily restricted in BHK-21 cells under the infection conditions used. This has been introduced in the extended lethal defection model by allowing new virus particles to enter *E* cells but not *I* cells. The inability of defective interfering particles to superinfect a cell was previously documented for poliovirus [75]. Coinfection of the same cell by the two LCMV versions was allowed, as suggested by the cells that included both the red and green markers (yellow cells, color merge), detected at time 0 between infections (Figures 7B and S1). Therefore, interference by genomes introduced by external LCMV particles requires the simultaneous infection by standard and interfering virus because superinfections appear to be highly restricted. The absence of reinfections implies that interference in a cell will be exerted essentially by interfering genomes generated in the same cell.

## Discussion

The continuous presence of a dynamic mutant spectrum in replicating RNA virus populations is a fundamental feature of RNA viruses that exerts a profound influence in their biology, and can jeopardize strategies to control viral disease [7], [8], [4]. Mutagenic nucleoside analogues are currently being investigated as antiviral agents because they can promote RNA virus extinction through lethal mutagenesis [[52], [50], [76], [51], [30], [33], [77], [78], [53], [73], [79]–[81]; reviewed in [11], [12], [9]]. Examination of the events that accompany FU-mediated extinction of LCMV [31], [33], [27], [34], [30], [32] led to the proposal of lethal defection as a mechanism of virus extinction [30]. The basic concept behind the lethal defection model is that some functionally altered genomes or gene products that are generated upon replication under enhanced mutagenesis interfere with replication of the more competent genomes, thus contributing to the replicative collapse of the entire viral population. Since its formulation, the lethal defection model has been supported by additional experimental results obtained with LCMV and other RNA viruses, as well as by theoretical studies [76], [36], [47], [33], [45], [30], [35], [49], [82]. The present investigation was undertaken to explore with LCMV, a system with a high endogenous production of defective genomes, the effect of the infecting viral dose on mutagen-induced virus extinction. The inhibitory activity of FU on infectious progeny production of LCMV was more pronounced in infections carried out at low than at high MOI, accompanied of a larger increase of mutant spectrum complexity in the progeny populations. While VSV, another negative strand RNA virus, behaved similarly to LCMV, a low MOI did not enhance either the inhibition by FU or the mutant spectrum complexity of FMDV and EMCV (compare Figures 1, 2 and Tables 1, 2). The results with VSV are in agreement with early quantifications of the effect of FU mutagenesis with VSV by Holand and collegues [77]. The difference observed may be influenced by the lower tendency of the positive strand RNA picornaviruses to establish interactions in *trans* as compared with negative strand RNA viruses [83], [84]. This is suggested by modest MOI-dependent progeny production acquired by EMCV upon passage in BHK-21 cells (Figure 6). Thus, passage history, related to production of, defective genomes, might be the relevant factor irrespective of the virus being of positive or negative sense RNA. The most abundant mutation types expected from FU-mediated mutagenesis are A→G and U→C transitions [52], [53], [80], [85], [50], and these are also those observed in the present experiments (Tables 1 and 2). In the case of LCMV, the most frequent transitions were A→G for gene L and U→C for Z (Table 1).

An approach to validate the experimental results using totally different tools has been to test whether the results are consistent with theoretical predictions. To this aim, we have developed an extended model inspired in previous versions of the lethal defection model developed by Manrubia and her colleagues [30], [46], [35]. The main features of this extended model are: i) cell coinfection is included; ii) an adjustable degree of *trans*-interaction is explicitly taken into account; iii) DI's are considered as a subclass of standard virus-dependent defectors; and iv) both replication and release of viral progeny from the cell can be affected by the mutation frequency. The model reproduces correctly the kinetics of progeny production and autointerference of LCMV observed at high MOI [86], [42], [87] (Figures 4 and 5). The model also reproduces the negative correlation between the inhibition of infectious virus production by FU and the MOI when the mutation rate is increased. In infections carried out at low initial MOI a larger number of infection rounds are needed to infect all the cells and, therefore, viruses can acquire a larger number of mutations per genome. In contrast, high MOI increases the number of initial infections, and reduces the number of replication rounds needed to reach the plateau of virus production. As a consequence, the viruses accumulate a lower average number of mutations, as reflected in the decrease in the mutation frequency (Table 3). An increase of the mutation frequency at low MOI favors the production of mutant and defector viruses, which in turn decrease the infectious progeny production. The lower progeny production of LCMV at high MOI may be due to interfering genomes and subsequent autointerference [86], [87]. The effect was noticed at late times presumably because interfering genomes had accumulated (Figure 1).

Results of infections with differentially labeled LCMVs indicated that while coinfections are allowed, superinfection is strongly restricted in BHK-21 cells (Figure 7). Restriction of superinfection was also observed with Junin virus in persistently infected BHK and Vero cells [88], [89]. Thus, interference and autointerference depend on the coinfection of cells by standard and defective genomes, and on the intracellular generation of mutant genomes, favored by FU mutagenesis.

Our experimental findings, supported by the extended lethal defection model, are relevant to the planning of lethal mutagenesis protocols because lower mutagenic intensities may be sufficient to achieve the extinction of viruses that are prone to produce defective genomes during their natural infectious cycle. It will be of interest to compare the effect of sequential and combination treatments involving a mutagenic agent and a non-mutagenic inhibitor [82], [85], [90], [76] on viruses prone to generate and tolerate a higher basal level of defective genomes (without an added mutagen) and on viruses restricted in defective genome generation and tolerance.

## Materials and Methods

### Cells, virus and infections

Growth of BHK-21 and Vero cells and virus infections were carried out as previously described [31], [91], [50], [27], [30], [33], [32]. LCMV ARM 53b is a triple plaque-purified clone from ARM CA 1371, passaged four times in BHK-21 cells. An LCMV virus stock (termed p0) was prepared by infecting BHK-21 monolayers (3×10^6^ cells in 100 mm-diammeter dishes) with 0.01 PFU of LCMV ARM 53b per cell. The p0 virus preparation was used for all the experiments. Other viruses employed are the Mudd-Summers strain of the Indiana serotype of VSV [92], clone C-S8c1 of FMDV [93], and the Rueckert strain of EMCV [94]. Procedures for LCMV infection have been described [33], [27], [32], [31], [30]. Briefly, semiconfluent monolayers of BHK-21 cells (3×10^6^ cells in 100 mm-diammeter dishes) were infected with LCMV ARM 53b p0 at different MOIs. After a 90 min adsorption period at 37°C and 7% CO_2_, the inocula were removed and the monolayers washed with Dulbecco modified Eagle medium (DMEM). The infected cultures were maintained in 10 ml of DMEM supplemented with 10% fetal calf serum (FCS), 2% L-glutamine, 0.52% glucose, 50 µg/ml gentamicin, either in the absence or the presence of FU (concentrations indicated in each experiment) at 37°C and 7% CO_2_. Supernatants of the infected cell cultures were sampled at different times postinfection (p.i.) and stored at −80°C. The infectivity of extracellular virus was determined by plaque assay on Vero cell monolayers as follows: 10^6^ cells per well in six-well dishes were infected by applying 300 µl of a dilution of the culture medium to be tested; after 90 minutes of adsorption at 37°C and 7% CO_2_, the monolayers were washed with DMEM and overlaid with 4 ml of DMEM 1% FCS, 1% DEAE-Dextran, 2% L-glutamine, 50 µg/ml gentamicin and 0,3% agar. After 7 days, cells were stained with 2% crystal violet in 2% formaldehyde, and viral plaques were counted. Supernatants from mock-infected cultures maintained in parallel were titrated to monitor the absence of viral contamination. No contamination (cytopathology or plaques) was detected in the control cultures throughout the experiments.

Infections of BHK-21 cells with FMDV, EMCV and VSV (6×10^6^ cells in 100 mm-diameter dishes) at different MOI were done as described [95], [93], [96]. The infected cultures were harvested 24 hours (h) p.i. and stored at −80°C. Virus infectivity was determined by plaque assay on BHK-21 cell monolayers as follows: 10^6^ cells in each of 35 mm-diammeter dishes were infected by applying 100 µl of serial dilutions of the culture medium; after 60 minutes of adsorption at 37°C and 7% CO_2_, the monolayers were washed with DMEM and overlaid with 4 ml of DMEM 1% FCS, 1% DEAE-Dextran, 50 µg/ml gentamicin and 0,5% agar. After 24 h, cells were stained with 2% crystal violet in 2% formaldehyde, and viral plaques were counted. Titers shown are the mean of at least three determinations. Again, mock-infected cultures were maintained in parallel to ascertain the absence of undesired viral infections.

To study the permissivity of BHK-21 cells to superinfection by LCMV, we used trisegmented versions of LCMV [74] engineered to express either the red fluorescent protein (RFP) (r3LCMV/RFP) or the green fluorescent protein (GFP) (r3LCMV/GFP). Semiconfluent monolayers of BHK-21 cells (1×10^6^ cells in 16 mm-diameter dishes) were infected with RFPrLCMV at different MOIs. Then the cell monolayers were washed, and the cells reinfected with GFPrLCMV at a MOI of 0.1 PFU/cell, at different times after the first infection. At 0 and 48 h after the second infection, the cells were fixed with 4% formaldehyde, washed two times with PBS, and cell nuclei were counterstained using DAPI for 10 minutes, and then maintained in PBS (100 µl/well) [74]. An inverted Axiovert S100 Zeiss fluorescence microscope was used to visualize the samples.

### Treatment with 5-fluorouracil

Preparation of FU stock solutions, determination of FU toxicity for BHK-21 cells, and procedures for viral infections in the presence of FU have been described [30], [50].

### RNA Extraction, reverse transcription–PCR, nucleotide sequencing, and determination of mutant spectrum complexity

RNA was extracted from supernatants of infected cultures using Trizol (Invitrogen), following the manufacturer's protocol. Triplicate RNA samples were amplified by reverse transcription–PCR (RT-PCR) using *RT transcriptor* (Roche) and *PFU* DNA polymerase (Promega). To ascertain that an excess of template was amplified and that the amount of template was not limiting during the RT-PCR amplification, a 1∶10 and 1∶100 dilution of the RNA was subjected to RT-PCR in parallel. Positive amplifications with the diluted template ensured that there was no limitation in the amount of viral RNA molecules as template for the RT-PCR amplification carried out with undiluted RNA [72]. cDNAs were purified with a Wizard PCR purification kit (Promega), pGEM-T Easy Vector (Promega), and cloned in *Escherichia coli* DH5α. cDNA from individual bacterial colonies was amplified with Templiphi (GE Healthcare). The oligonucleotides used for amplification of two LCMV genomic regions were: L3654 (forward 5′- AGT TTA AGA ACC CTT CCC GC - 3′) and L4260 (reverse 5′ CGA GAC ACC TTG GGA GTT GTG C - 3′) for the polymerase region; L7 (forward 5′- GGG GAT CCT AGG CGT TTA GT- 3′) and L402 (reverse 5′- GGA ACC GCA CGT CGC CCA ACG CAC - 3′) for the Z gene. The number in the primer designation corresponds to the 5′ nucleotide position, and refers to the consensus genomic RNA sequence determined previously [31] [GenBank accession numbers AY847351(L) and AY847350(S)]. The oligonucleotides used for amplification of a fragment of the polymerase gene of VSV, FMDV and EMCV were L5902F (forward 5′- GCAAGTGATTTAGCTCGGATT - 3′; residues 5903-5923) and L6569R (reverse 5′- GGTGGTTATTCCATTTTTCG - 3′ residues 6569-6550) for VSV; 3DR3 (forward 5′- CAAAGATGTCTGCGGAGGACAA - 3′; residues 6800-6821) and AV3 (reverse 5′- TTCATGGCATCGCTGCAGTGG - 3′; residues 7370-7350) for FMDV; EM67D (forward 5′- CAGCCACCCTGATCCCGTTTGC - 3′; residues 6718-6739) and EM73R (reverse 5′- CTTATATCCTGTCTTTGCGAGGC - 3′; residues 7403-7381) for EMCV. Nucleotide positions correspond to genomic RNAs that have been previously described [97]–[99]. To characterize the mutant spectra of the viral quasispecies, 20 to 48 clones per sample were sequenced (7,800 to 32,000 nt per population). The complexity of the quasispecies was characterized by means of two parameters: (*i*) mutation frequency (average number of mutations per nucleotide sequenced, relative to the consensus sequence of the corresponding mutant spectrum) and (*ii*) Shannon entropy, a measure of the proportion of genomes that are different in the mutant spectrum of the quasispecies. To calculate the mutation frequency, repeated mutations found in the same quasispecies were counted only once (minimum mutation frequency). Shannon entropy was calculated with the formula: Sn = −[Σ_i_ (*p*_i_×ln *p*_i_)]/ln *N*, in which *p*_i_ is the frequency of each sequence in the quasispecies and *N* is the total number of sequences compared. A Shannon entropy value of 0 indicates that all of the sequences are identical whereas a value of 1 indicates that each genome differs from the others in its nucleotide sequence [100].

### LCMV RNA quantification

RT and real-time quantitative PCR were carried out using the Light Cycler DNA Master SYBR Green I kit (Roche), according to the manufacturer's instructions. The polymerase region was amplified with primers L4183 (forward 5′ - ATC GAG GCC ACA CTG ATC TT – 3′) and L4260 (same numbering for primer location as described for RT-PCR amplification). An LCMV RNA fragment spanning nucleotides 3662 to 4268 was used as standard. This was obtained as a runoff transcript from a molecular DNA clone encoding the polymerase-coding region in the genomic sense, cloned into pGem-T Easy Vector (Promega). The denaturation curve of the amplified DNAs was determined to monitor the specificity of the amplification. Negative controls (without template RNA) were run in parallel with each amplification reaction. Each value of the amount of LCMV RNA is the average of at least three determinations. This procedure for LMCV RNA quantification was used previously [33], [30].

### Statistical analyses

#### General linear model

To study the interaction between the MOI and the inhibition of virus progeny production by FU for different viruses, a multivariate general linear model was used according to the formula: 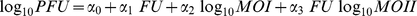
in which FU is the concentration of FU, MOI is the multiplicity of infection, 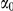 is the ordinate at the origin, 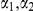 are the regression coefficients that express the variation of log PFU with respect to each of the independent variables, and 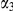 is the coefficient that expresses the interaction among different variables. A positive interaction coefficient α_3_ indicates that one variable accentuates the effect of the other; in this case, an increase of the MOI would increase the inhibitory effect of FU. A negative interaction coefficient indicates that one variable decreases the effect of the other; in this case, an increase in the MOI would decrease the inhibitory effect of FU.

## Supporting Information

Figure S1 **LCMV coinfections and reinfections of BHK-21 cells.** BHK-21 cells were infected with RFPrLCMV at MOI of 0.001 (A and B), 0.01 (C and D). After 0, 4, 8, 12 or 24 h p.i. cells were re-infected with GFPrLCMV at a MOI of 0.1 PFU/cell. Panels depict the infected cells at 0 (A and C) and 48 (B and D) h after the GFPrLCMV infection (nucleus stained with DAPI, cells expression, RFP, GFP and merged image of the three panels). Magnification is 20-fold. Procedures are detailed in Materials and Methods and conclusions in the main text.
(TIF)

Figure S2 **LCMV coinfections and reinfections of BHK-21 cells.** BHK-21 cells were infected with RFPrLCMV at MOI of 0.1 (E and F), 1 (G and H). After 0, 4, 8, 12 or 24 h p.i. cells were re-infected with GFPrLCMV at a MOI of 0.1 PFU/cell. Panels depict the infected cells at 0 (E and G) and 48 (F and H) h after the GFPrLCMV infection (nucleus stained with DAPI, cells expression, RFP, GFP and merged image of the three panels). Magnification is 20-fold. Procedures are detailed in Materials and Methods and conclusions in the main text.
(TIF)

Figure S3 **LCMV coinfections and reinfections of BHK-21 cells.** BHK-21 cells were infected with RFPrLCMV at MOI of 10 PFU/cell (I and J) and MOCK (K and L). After 0, 4, 8, 12 or 24 h p.i. cells were re-infected with GFPrLCMV at a MOI of 0.1 PFU/cell. Panels depict the infected cells at 0 (I and K) and 48 (J and L) h after the GFPrLCMV infection (nucleus stained with DAPI, cells expression, RFP, GFP and merged image of the three panels). Magnification is 20-fold. Procedures are detailed in Materials and Methods and conclusions in the main text.
(TIF)

Table S1 **Parameters used in the model.**
(DOCX)

Text S1 **Genoset-associated functions.**
(DOCX)
