# Supplementary figures and images for: Mutagenesis-Mediated Virus Extinction: Virus-Dependent Effect of Viral Load on Sensitivity to Lethal Defection
Source: PLoS One. 2012 Mar 19;7(3):e32550. doi: 10.1371/journal.pone.0032550 (PMC3307711; doi:10.1371/journal.pone.0032550)

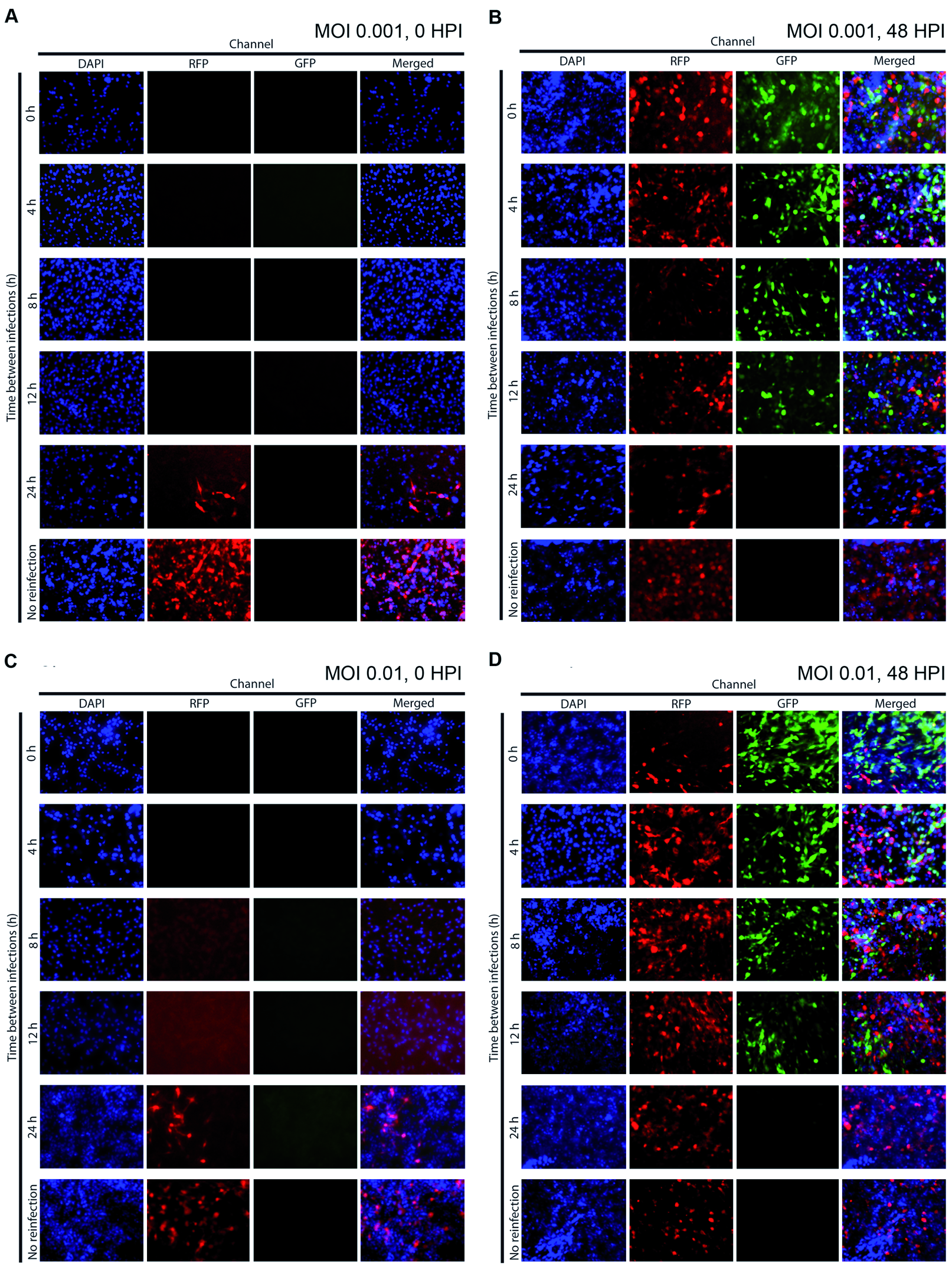

Supplement: Figure S1 — LCMV coinfections and reinfections of BHK-21 cells. BHK-21 cells were infected with RFPrLCMV at MOI of 0.001 (A and B), 0.01 (C and D). After 0, 4, 8, 12 or 24 h p.i. cells were re-infected with GFPrLCMV at a MOI of 0.1 PFU/cell. Panels depict the infected cells at 0 (A and C) and 48 (B and D) h after the GFPrLCMV infection (nucleus stained with DAPI, cells expression, RFP, GFP and merged image of the three panels). Magnification is 20-fold. Procedures are detailed in Materials and Methods and conclusions in the main text. (TIF) [file pone.0032550.s001.tif]

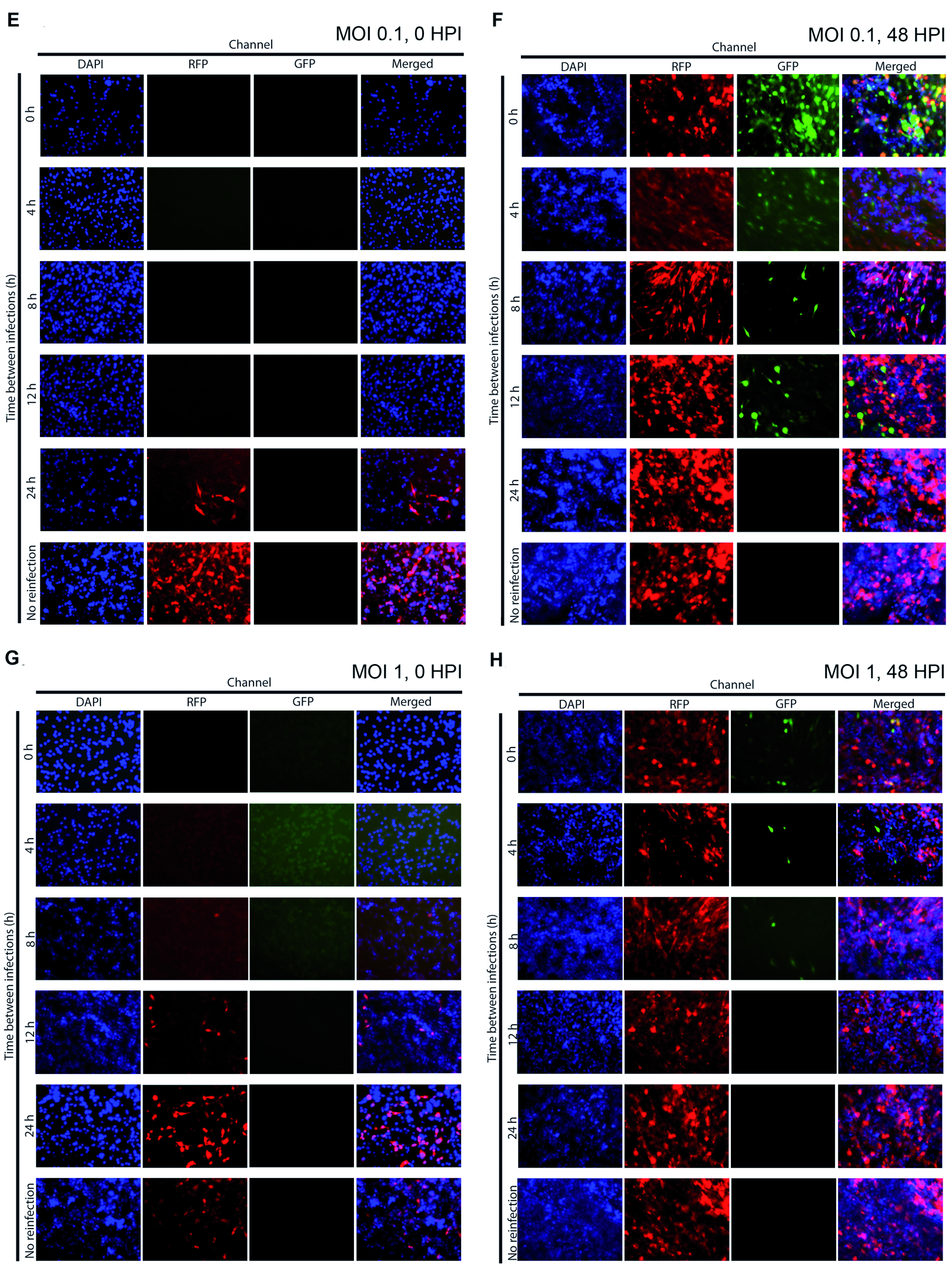

Supplement: Figure S2 — LCMV coinfections and reinfections of BHK-21 cells. BHK-21 cells were infected with RFPrLCMV at MOI of 0.1 (E and F), 1 (G and H). After 0, 4, 8, 12 or 24 h p.i. cells were re-infected with GFPrLCMV at a MOI of 0.1 PFU/cell. Panels depict the infected cells at 0 (E and G) and 48 (F and H) h after the GFPrLCMV infection (nucleus stained with DAPI, cells expression, RFP, GFP and merged image of the three panels). Magnification is 20-fold. Procedures are detailed in Materials and Methods and conclusions in the main text. (TIF) [file pone.0032550.s002.tif]

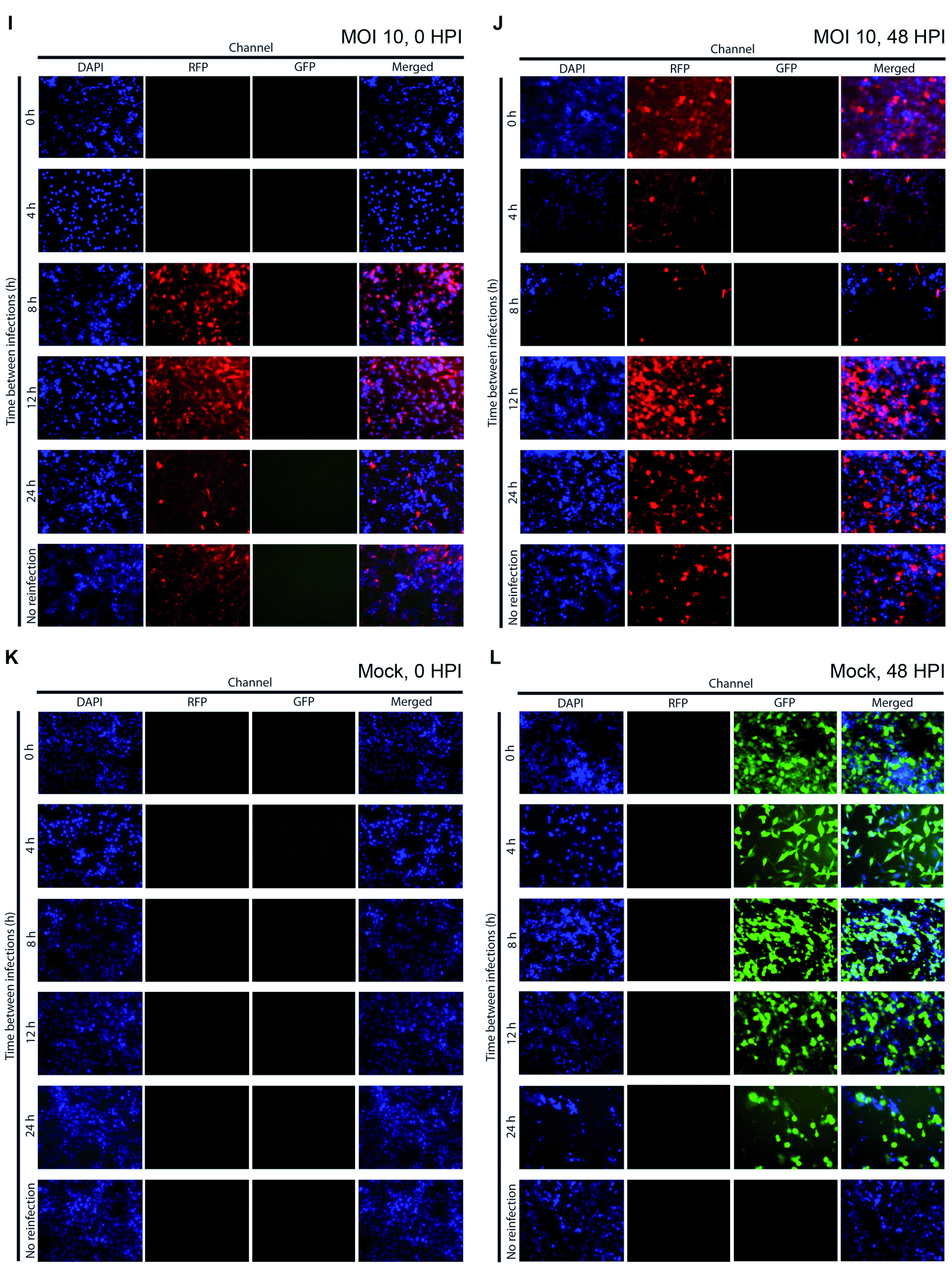

Supplement: Figure S3 — LCMV coinfections and reinfections of BHK-21 cells. BHK-21 cells were infected with RFPrLCMV at MOI of 10 PFU/cell (I and J) and MOCK (K and L). After 0, 4, 8, 12 or 24 h p.i. cells were re-infected with GFPrLCMV at a MOI of 0.1 PFU/cell. Panels depict the infected cells at 0 (I and K) and 48 (J and L) h after the GFPrLCMV infection (nucleus stained with DAPI, cells expression, RFP, GFP and merged image of the three panels). Magnification is 20-fold. Procedures are detailed in Materials and Methods and conclusions in the main text. (TIF) [file pone.0032550.s003.tif]
