# Supplementary material for: Mutagenesis-Mediated Virus Extinction: Virus-Dependent Effect of Viral Load on Sensitivity to Lethal Defection
Source: PLoS One. 2012 Mar 19;7(3):e32550. doi: 10.1371/journal.pone.0032550 (PMC3307711; doi:10.1371/journal.pone.0032550)
Supplement: Table S1 — Parameters used in the model. (DOCX) [file pone.0032550.s004.docx]

**Table S1. Parameters used in the model**

| **Parameter** | **Meaning** | **Value^a^** |
| --- | --- | --- |
| **Extracellular Dynamics** | | |
| *β* | Virus Internalization rate | 0.2 |
| *k_e_* | Exposed-to-infected transition rate | 2 |
| *k_i_* | Viral production rate | 2 |
| *u* | Virus degradation rate | 1 |
| *maxVirusxCell* | Maximum number of infecting virus allowed per cell | 3 |
| **Intracellular dynamics** | | |
| *nS* | S genoset length | 500 |
| *nR* | R genoset length | 500 |
| *nP* | P genoset length | 500 |
| *nD* | D genoset length | 50 |
| *w_0_* | Number of copies produced by the replication of the reference viral genome. | 50 |
| *cisR* | Degree of *cis*-acting of the genoset R | 0.25 - 1 |
| *transR* | Degree of *trans*-acting of the genoset R | 0.75 - 0 |
| *RepAdv* | Replicative advantage of DI inside the cell | 2.5 |
| *maxBsize* | Maximum number of viral particles produced by a cell per replication cycle | 20 |
| *sS* | Selective coefficient for genoset *S* | 0.1 |
| *uR* | Mutation threshold for the activity of genoset R | 3 |
| *uP* | Mutation threshold for the activity of genoset P | 5 |
| *U* | Average number of mutations per genome and replication rate | 0.15-7.5 |
| *m* | Mutation rate per position = *U/(nS+nR+nP+nD)* | 10^-4^-5·10^-3^ |

^a^ The numerical values of the parameters have been chosen arbitrarily, based on experimental values when available [*U*, m according to mutation rates values [6,7,8]] and *uR*, *uP* according to estimates of the error threshold for RNA viruses [9,10,11,12]. However, the robustness of the model (independence of the results of the simulation when parameter values are varied) was tested by performing simulations in which values for parameters *nS*, *nR*, *nP*, *nQ*, *w_o_*, *RepAdv*, *maxBsize*, *sS*, *uR* and *uP* were altered 2 to 10-fold (the interval depending on the parameter), relative to the listed values. Although parameter variation modified quantitatively the results of the model, the latter did not vary qualitatively. In particular, a greater inhibition of infectious progeny production and higher mutation frequency at low MOI was predicted over the range or parameter values tested. Thus, the model reproduces qualitatively the experimental results in a robust way. A detailed analysis of the effect of the parameter space, as well as the effect of more complex and realistic genoset-associated functions, on the quantitative results of the model will be published elsewhere.
