# Supplementary material for: Mutagenesis-Mediated Virus Extinction: Virus-Dependent Effect of Viral Load on Sensitivity to Lethal Defection
Source: PLoS One. 2012 Mar 19;7(3):e32550. doi: 10.1371/journal.pone.0032550 (PMC3307711; doi:10.1371/journal.pone.0032550)
Supplement: Text S1 — Genoset-associated functions. (DOCX) [file pone.0032550.s005.docx]

**Supporting Information S1**

***Genoset-associated functions:***

*S Genoset:*

The numerical value (s_i_) assigned to this genoset in any individual viral genome is taken as$s_{i}=(1-sS)^{i}$, where sS is a selective coefficient that determines the effect of a mutation. The lower the value of s_i_, the lower the ability of genome *i* to be replicated either by the proteins encoded by genome *i*, or by the proteins encoded by other viral genomes in the same cell. Although not considered in this model for simplicity, the effect of a lethal mutation would be to render *s_i_* = 0. The length of the S genoset is n_S_.

*R Genoset:*

The effect of genoset *R* for genome *i* (*r_i_*) is determined by a truncated function as follows:

$$\left\{\begin{matrix} r_{i}=1\text{ if }i\leq uR \\ r_{i}=0\text{ if }i<uR \end{matrix}\right.$$

where *uR* is a mutation threshold beyond which genome *i* can not contribute to the replicating genome pool inside the cell (due to excess of mutation). A genome which acts as a defector for replication is defined as having *r_i_* = 0, it does not give rise to the functional proteins needed for viral replication, but it can use the proteins produced from other viral genomes for its own replication, therefore interfering with the standard virus replication. The length of the *R* genoset is *n_R_.*

*P Genoset:*

As for R, the effect of genoset *P* for genome *i* (*p_i_*) is determined by a truncated function as follows:

$$\left\{\begin{matrix} p_{i}=1\text{ if }i\leq uP \\ p_{i}=0\text{if }i<uP \end{matrix}\right.$$

where *uP* is a mutation threshold beyond which genome *i* can not contribute to the production and release of virus from the cells. As for the case of the *R* genoset, a genome with *p_i_* = 0 is a defector for viral production because it does not contribute with functional proteins to the formation and release of virions. However, this class of defectors can be encapsidated by proteins generated from other genomes with viable *P* genosets. The length of the R genoset is *n_P_.*

*D Genoset:*

The probability of a viral genome to become a DI during replication will depend on the genoset length n_D_ according to $1-(1-m)^{nD}$where m is the mutation rate per nucleotide position $m=\frac{U}{\sum_{i} n_{i}}$and n_D_ is the length of the D genoset. Usually, DI are produced by deletions, or other genomic alterations that confer the DI a replicative advantage over the standard virus. This advantage allows the DI to persist in time, despite its inability to complete an infectious cycle by itself, by establishing host-parasite-like dynamics with standard virus, as suggested by several computational and mathematical models [1-5].

**References**

1. Thompson KA, Yin J (2010) Population dynamics of an RNA virus and its defective interfering particles in passage cultures. Virol J 7: 257.

2. Bangham CR, Kirkwood TB (1993) Defective interfering particles and virus evolution. Trends Microbiol 1: 260-264.

3. Bangham CR, Kirkwood TB (1990) Defective interfering particles: effects in modulating virus growth and persistence. Virology 179: 821-826.

4. Kirkwood TB, Bangham CR (1994) Cycles, chaos, and evolution in virus cultures: a model of defective interfering particles. Proc Natl Acad Sci USA 91: 8685-8689.

5. Stauffer Thompson KA, Rempala GA, Yin J (2009) Multiple-hit inhibition of infection by defective interfering particles. J Gen Virol 90: 888-899.
